# Effects of different exercise types on visceral fat in young individuals with obesity aged 6–24 years old: A systematic review and meta-analysis

**DOI:** 10.3389/fphys.2022.987804

**Published:** 2022-09-26

**Authors:** Rong Wang, Xiaoyuan Zhang, Hong Ren, Huixuan Zhou, Yaqing Yuan, Yunlong Chai, Xiao Hou

**Affiliations:** ^1^ School of Sport Science, Beijing Sport University, Beijing, China; ^2^ Department of Physical Education, Peking University, Beijing, China; ^3^ College of Sports and Health, Shandong Sport University, Shandong, China

**Keywords:** exercise, visceral fat (VFA), young individuals, adolescents, obesity, overweight, meta-analysis

## Abstract

**Introduction:** The prevalence of pediatric obesity remains high all over the world. Various exercise interventions have been applied to decrease the visceral fat in young individuals with obesity. But the evidence remains controversial on the effect of the exercise on visceral fat. Moreover, it is unclear which type of the exercise is the most effective for young individuals with overweight or obesity to reduce visceral fat.

**Objective:** The objective of this review and meta-analysis is to evaluate and compare the effectiveness of different exercise interventions on visceral fat in young individuals with overweight or obesity.

**Methods:** Four databases consisting of PubMed, Web of Science, EBSCO, and Cochrane Library were searched prior to May 2022. Fifteen studies with a total of 30 data points involving 1,134 participants were included in this meta-analysis. And the interventions were limited to 4 exercise types [i.e., aerobic exercise (AE), resistance exercise (RE), aerobic exercise combined with resistance exercise (CE), and high-intensity interval training (HIIT)].

**Data Synthesis:** The results showed that AE (Standardized Mean Difference = −0.32; 95% CI = −0.50 to −0.13; *p* = 0.0007; I^2^ = 37%) and HIIT (SMD = −0.59; 95% CI = −0.87 to −0.31; *p* < 0.0001; I^2^ = 0%) had a significant reduction effect on visceral fat. And the effect of HIIT seemed better than AE. However, RE (SMD = −0.58; 95% CI = −1.34 to 0.17; *p* = 0.13; I^2^ = 76%) and CE (SMD = −0.21; 95% CI = −0.81 to 0.38; *p* = 0.48; I^2^ = 63%) had a non-significant effect on visceral fat decline. Additionally, compared with the control group, exercise interventions had a significant effect on reducing visceral fat in adolescents (SMD = −0.54; 95% CI = −0.82 to −0.26; *p* = 0.0001; I^2^ = 64%) and young adults (SMD = −0.42; 95% CI = −0.69 to −0.15; *p* = 0.003; I^2^ = 0%) rather than children (SMD = −0.15; 95% CI = −0.32 to 0.02; *p* = 0.08; I^2^ = 0%). And the gender-based subgroup analysis indicated that the effectiveness of the exercise on the reduction of visceral fat was more significant in males (SMD = −1.27; 95% CI = −1.67 to −0.87; *p* < 0.00001; I^2^ = 0%) than that in females (SMD = −0.31; 95% CI = −0.48 to −0.14; *p* = 0.0004; I^2^ = 0%).

**Conclusion:** This review and meta-analysis demonstrates that exercise interventions are efficient to decrease visceral fat in adolescents (12–18 years old) and young adults (18–24 years old). Among different exercise types, AE and HIIT are helpful for young individuals with overweight or obesity to reduce visceral fat and HIIT appears to be the most effective exercise intervention. In addition, the effect of exercise interventions on the consumption of visceral fat is more significant in males than that in females.

**Systematic Review Registration:** [http://www.crd.york.ac.uk/PROSPERO], identifier [CRD42022310878].

## Introduction

It is widely known that the prevalence of overweight and obesity trends towards the younger ages in recent years. In 2020, World Health Organization (WHO) announced that 38.9 million children under the age of 5 years old are overweight (WHO, 2021). Overweight and obese young individuals (e.g., children, adolescents, or young adults) are more prone to develop diabetes and cardiovascular diseases and to maintain obesity in older-aged adulthood (Wang and Lim, 2012; Al-Sulaiti et al., 2019; WHO, 2020). The sustained effect of obesity from childhood to adulthood makes a variety of diseases more apparent in the adult period, such as heart disease, stroke, diabetes, osteoarthritis, endometrial, breast, and colon (WHO, 2020). In addition to the threat to physical health, obesity can also impair young individuals’ mental soundness. It has been reported that overweight or obese adolescents have a higher prevalence of depression and anxiety symptoms than adolescents who are not obese (Wang et al., 2019). Those obese children or adolescents frequently fall into a vicious cycle between distress, damage and distortion of self-image, maintenance and deterioration of pain (Pervanidou and Chrousos, 2011).

Abnormal or excessive fat accumulation including subcutaneous fat and visceral fat can lead to obesity at a young stage, which is identified as an important risk factor for health in the full life cycle. Various studies have demonstrated that, compared to total body fat mass or subcutaneous fat, excessive visceral fat should be more blamed for several chronic metabolic diseases (e.g., insulin resistance and hypertension) (Fox et al., 2007; Gastaldelli et al., 2007; Suliga, 2009; Karlsson et al., 2019). Tadokoro et al. have demonstrated that boys aged 15–16 years old with more accumulated visceral fat were detected with a higher level of atherosclerosis, liver dysfunction, hyperlipidemia, and hyperinsulinemia (Tadokoro et al., 2010). It has been reported that visceral fat thickness is strongly correlated to the elastic properties of the abdominal aorta (Polat et al., 2008). Hence, the extra visceral fat may be a hidden danger for young people with visceral obesity to develop certain cardiovascular diseases. Besides that, the excessive accumulation of visceral fat can lead to the decreased immunity (de Heredia et al., 2012), the high occurrence of chronic obstructive pulmonary disease (Furutate et al., 2011), and even some common cancers such as colorectal cancer (Larsson and Wolk, 2007; Tsang et al., 2009; Kang et al., 2010) and esophageal cancer (Elliott and Reynolds, 2021). Therefore, it is necessary to pay more attention to excessive visceral fat accumulation rather than total body fat or subcutaneous fat and to find effective interventions to reduce visceral fat in young individuals with overweight or obesity.

Exercise interventions are the effective and cost-efficient strategies to optimize the body composition of individuals at a younger age (Stoner et al., 2016; Kim et al., 2020; Bogataj et al., 2021; Ouerghi et al., 2022). However, the evidence remains controversial on the effect of the exercise on visceral fat. For example, Tadokoro et al. have found that even in healthy high school students who do regular exercises, visceral obesity also occurs in 9.6% of participants (Tadokoro et al., 2010). In a randomized controlled trial (RCT) that examined the effect of the exercise on visceral fat, although subjects were instructed with exercise interventions in the experimental group, visceral fat did not decrease significantly in obese young individuals (Alberga et al., 2015).

In addition, it is widely reported that various exercise types are beneficial for young individuals with overweight or obesity to decrease visceral fat. Specifically, some researchers have suggested that aerobic training can effectively reduce visceral fat mass in obese adolescents (Gutin et al., 2002; Foschini et al., 2010; Alberga et al., 2013). A case in point showed that a 12-week aerobic exercise (AE) without calorie restriction (30 min/session, 4 sessions/week) was effective for the decline of visceral adipose tissue (VAT) (van der Heijden et al., 2010). While, many studies have also confirmed the role of resistance training in the decrease of visceral fat (Lee et al., 2012; Shultz et al., 2014; Lee et al., 2020). For example, a non-contact boxing-oriented intervention followed by the progressive resistance exercise (RE) has been regarded as an active exercise program to help obese young people decrease their visceral fat thickness (Shultz et al., 2014). Several studies have illustrated that aerobic combined with resistance exercise (CE) has a more obvious effect on the decline of visceral fat in young people with obesity than the isolated AE or the isolated RE (Damaso et al., 2014; Jung et al., 2019). High-intensity interval training (HIIT), as a novel exercise training pattern, has also been proposed to burn visceral fat and optimize body composition in obese individuals (Maillard et al., 2018; Mendelson et al., 2022). It has been revealed that HIIT can produce a variety of health benefits in school-aged individuals with obesity, such as reducing total body mass and visceral fat (Zhang et al., 2017), improving cardiovascular fitness (Costigan et al., 2015; Kong et al., 2016), increasing muscle strength and peak oxygen uptake (Abarzua et al., 2019). Although different types of exercise interventions have been suggested conducive to the reduction of visceral fat in obese or overweight young individuals. It is still unclear which type of the exercise is the most effective intervention for decreasing visceral fat. Considering that obesity at a young age has a lasting harmful effect on adulthood, it is necessary to examine studies related to obese or overweight young individuals to compare the effectiveness of different types of exercise interventions on visceral fat.

The purpose of our study is to evaluate and compare the effect of exercise interventions (AE, RE, CE, and HIIT) on visceral fat in overweight or obese young individuals through a meta-analysis from a more comprehensive and systematic perspective.

## Methods

### Search strategy and study selection

This systematic review and meta-analysis was performed according to the Preferred Reporting Items for Systematic Reviews and Meta-Analyses (PRISMA) guidelines. The protocol was registered on the international prospective register of systematic reviews (http://www.crd.york.ac.uk/PROSPERO), registration number: CRD42022310878. No similar systematic review protocol exists.

Four databases, namely, PubMed (1988–2022), Web of Science (2003–2022), EBSCO (1997–2022), and Cochrane Library (1991–2022) were searched using the following keywords: [“exercise” OR “training” OR “aerobic” OR “resistance” OR “aerobic plus resistance” OR “combined training” OR “HIIT” OR “sprint interval training (SIT)” OR “repeated sprint interval training (RST)”] AND (“body composition” OR “visceral fat” OR “VAT” OR “visceral adipose” OR “abdominal fat” OR “abdominal adipose”) AND (“obesity” OR “obese” OR “overweight”) AND (“adolescent” OR “youth” OR “children” OR “teenagers” OR “young”). Two authors (R.W. and X.Z.) screened and extracted relevant studies independently through reviewing all titles, abstracts, and full-text articles. Any disagreement was resolved by discussion until consensus was reached or by consulting a third arbitrator (X.H.).

### Inclusion criteria

According to the PICOS principle (patient/population, intervention, comparison/control, outcome and study design), trials were eligible for inclusion if they met the criteria as follows: 1) participants were defined as overweight [body mass index (BMI) ≥ 85th percentile or BMI ≥25 kg/m^2^] or obese (BMI ≥95th percentile or BMI ≥30 kg/m^2^) young individuals aged 6–24 years old, participants with body fat percentage (BFP)≥30% for females and≥25% for males, or participants whose triceps skinfold thickness (TSF) was greater than the 85th percentile; 2) the exercise interventions were limited to AE, RE, CE and HIIT; 3) no exercise intervention was applied in the control group in the same duration as the experimental group; 4) the outcomes of the trial included the common indices reflecting visceral fat: visceral fat mass, VAT, the thickness of abdominal visceral fat, abdominal visceral fat area, the prevalence of visceral obesity or abdominal adipose; 5) only the trials designed as RCTs were covered; and 6) the selected articles were peer-reviewed publications written in English.

### Exclusion criteria

Trials were excluded when they met any of the following exclusion criteria: 1) reviews, abstracts, case reports, observational studies, non-peer-reviewed articles, such as dissertations or conference posters; 2) participants have been diagnosed with mental disorders or eating disorders; 3) participants were pregnant women or individuals with orthopedic/neurological disorders that limited the exercise ability; 4) the specific types of exercise interventions couldn’t be identified; 5) the experimental group was intervened with the exercise and drugs; 6) the control group received any drug treatment; 7) incomplete data or inadequate descriptions of research methods in the study.

### Quality assessment

Two independent authors used the Cochrane Collaboration tool to evaluate the quality of the included studies according to the seven domain biases as follows: 1) Random sequence generation (selection bias); 2) allocation concealment (selection bias); 3) blinding of participants and personnel (performance bias); 4) blinding of outcome assessment (detection bias); 5) incomplete outcome data (attrition bias); 6) selective reporting (reporting bias); and 7) other bias (Higgins et al., 2011). Three grades of high, low, or unclear bias were labeled for each included study. Any disagreement was resolved by discussion until consensus was reached or by consulting a third arbitrator (X.H.).

### Data extraction

Two authors independently extracted the relevant data from each included study as the following: author(s), publication year, country/region, subjects’ characteristics (e.g., age, BMI, gender), sample size, intervention (e.g., intensity, frequency, duration, exercise type), reported outcomes (e.g., VAT, the thickness of abdominal visceral fat, and the abdominal visceral fat area).

### Meta-analysis

The authors used the Review Manager software (Review Manager 5.3; The Nordic Cochrane Center, The Cochrane Collaboration) to perform this meta-analysis. The outcomes in the meta-analysis included visceral fat mass (g, kg, L, cm^3^), the thickness of abdominal visceral fat (cm, defined as the distance between the inner wall of the abdominal cavity and the anterior wall of the aorta), or visceral fat area (quantified in cm^2^ at the level between the 4^th^ and 5^th^ lumbar vertebra). In accordance with the Cochrane Handbook for Systematic Reviews (Higgins and Green, 2008), either post-intervention values (Mean _post−intervention_ ± SD _post−intervention_) of the outcome or changes from baseline (Mean _of changes_ ± SD _of changes_) could be used to calculate the summary statistic value.

If studies reported SE instead of SD, we would calculate SD using the formula: SD = SE × √N (Higgins et al., 2011). If studies only presented CI, the SD would be calculated by the formula “√*N* × (*l*_upper_-*l*_lower_)/c”, in which *l*_upper_ and *l*_lower_, respectively, represented the upper and lower limits of the CI. And c was a constant which depended on the CI and the sample size (Huedo-Medina et al., 2006). If articles presented the outcomes using figures, the GetData Graph Digitizer 2.26 would be used to extract the relevant data. If the mean and SD were not presented in the articles, we would contact the authors by email and if the authors didn’t reply, the articles would be excluded.

The I^2^ index was used to assess the heterogeneity among studies. Low, moderate, high and very high heterogeneity were identified when I^2^ ≤ 25%, I^2^ ≤ 50% and >25%, I^2^ ≤ 75% and >50%, I^2^ > 75% respectively (Huedo-Medina et al., 2006). If the heterogeneity was low or moderate, a fixed-effect model would be used. While, if the heterogeneity was high or very high, a random-effect model would be applied (Hou et al., 2021). Considering that, in this meta-analysis, the units (e.g., g, kg, L, cm, cm^2^, cm³) of outcomes reflecting visceral fat were different. We performed the Standardized Mean Difference (SMD) to analyze the compositive effects. The subgroup analysis was used to analyze the effectiveness of different exercise types (AE, RE, CE, and HIIT) on visceral fat in young individuals with overweight or obesity. And the other two subgroup analyses based on participants’ age and gender were also performed. When I^2^ > 50%, possible publication bias was assessed by examining the asymmetry of funnel plots or using Egger’s test (Egger et al., 1997). The level of significance was set at *p* < 0.05.

## Results

### Search result

**FIGURE 1:**
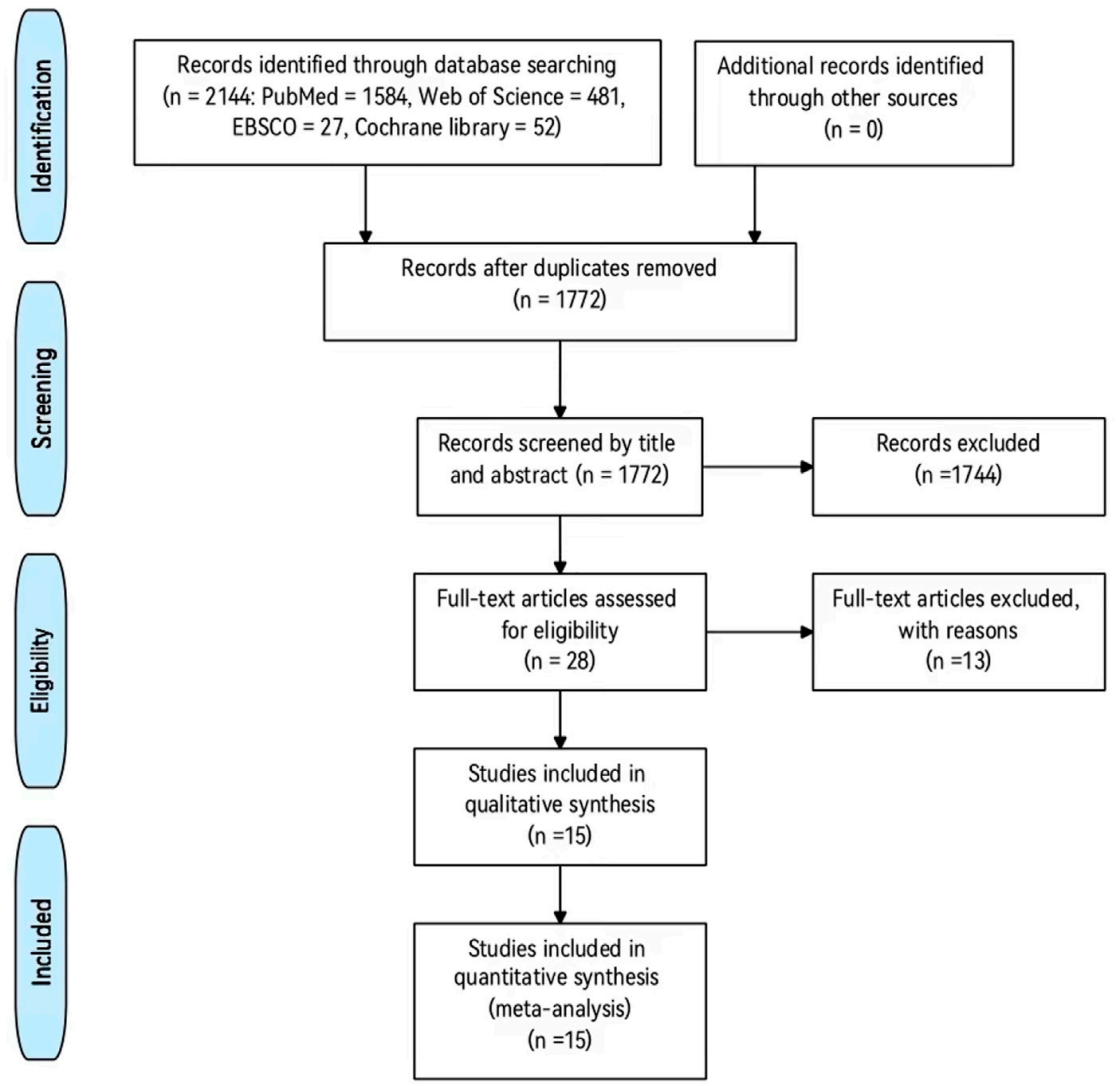
The flowchart of the search procedure.

The flowchart in Figure 1 presents the search procedure. Based on our preliminary search of four databases, a total of 2,144 records were identified. 1772 records of them remained after excluding duplicates. 28 potentially eligible articles remained after screening titles and abstracts. 15 articles met the inclusion after reviewing the full-text articles. The 15 articles were pooled in this meta-analysis and a total of 30 data points (involving 1,134 participants) were included.

### Characteristics of included studies

Table 1 shows the basic characteristics of the included articles. The subjects in all studies were children aged 6–12 years old, adolescents aged 12–18 years old, and young adults aged 18–24 years old. Among these studies, the subjects in three articles were children (Barbeau et al., 2007; Saelens et al., 2011; Davis et al., 2012). The subjects in eight articles were adolescents (Mitchell et al., 2002; Davis et al., 2011; Lee et al., 2012; Lee et al., 2013; Alberga et al., 2015; Monteiro et al., 2015; Staiano et al., 2017; Cao et al., 2022). The subjects in three articles were young adults (Zhang et al., 2017; Tong et al., 2018; Zhang et al., 2021). And one article included both children and adolescents (Dias et al., 2018). For the gender of the recruited subjects, seven articles only recruited females. Two articles only recruited males. And six articles recruited both males and females as subjects.

**TABLE 1:** Characteristics of included studies.

No.	Authors (publication year)	Country	Subjects’ characteristic	Sample size	Intensity	Frequency	Duration	Exercise type	Diet control	Outcome
1	Lee et al. (2013)	America	adolescents (age:12–18 years; BMI ≥95th percentile; gender: female)	AE: 16 RE: 16 CON: 12	AE: the former 1 week: ∼50% VO_2peak_×40 min the latter 11 weeks: 60∼75% VO_2peak_×60 min RE: the former 4 weeks: 60% *1RM; 8 ∼ 12 repetitions/set; 1∼2 sets the latter 8 weeks: 60% *1RM; 8∼12 repetitions/set; 2 sets until fatigue	AE: 60 min/session; 3 sessions/week RE: 60 min/session; 3 sessions/week	3 months	AE: treadmills and/or ellipticals RE: 10 whole body exercises using weight machines (leg press, leg extension, leg flexion, chest press, latissimus pulldown, seated row, bicep curl, and triceps extension) + push-ups and sit-ups CON: no exercise	AE and RE and CON: all groups were asked to follow a weight maintenance diet (55–60% carbohydrate, 15–20% protein, and 25–30% fat)	VAT area (cm^2^)
2	Monteiro et al. (2015)	Brazil	adolescents (age:11–17 years; BMI ≥95th percentile; gender: male and female)	AE: 18 CE: 14 CON: 16	AE: 65∼85% VO_2peak_ CE: AE + 55∼75% *10RM	AE: 50 min/session; 3 sessions/week CE: 60 min/session (30 min AE + 30 min RE); 3 sessions/week	20 weeks	AE: walking and running CE: AE + resistance training using weight machines (leg press, low rowing, bench press, squat rack, seated lat pull-down, leg curl, arm curl, seated chest fly, triceps, leg extension, sit up, and supine trunk extension) CON: no exercise	AE and CE and CON: none	the thickness of visceral fat (cm)
3	Alberga et al. (2015)	Canada	adolescents (age:14–18 years; BMI ≥85th percentile; gender: male and female)	AE: 54 RE: 55 CE: 55 CON: 57	AE: 70∼85% HR_max_ RE: ML (6–15 repetitions; 2–3 sets) CE: AE + RE	AE: 20∼40 min/session; 4 sessions/week RE: 4 sessions/week CE: 4 sessions/week	22 weeks	AE: treadmill, cycle ergometer or elliptical machine RE: whole-body exercises using resistance machines CE: AE + RE CON: no exercise	AE and RE and CE and CON: all groups received dietary counseling designed to promote healthy eating with a maximum daily energy deficit of 250 kcal	VAT (cm^2^)
4	Lee et al. (2012)	America	adolescents (age:12–18 years; BMI ≥95th percentile; gender: male)	AE: 16 RE: 16 CON: 13	AE: the former 1 week: ∼50% VO_2peak_×40 min the latter 11 weeks: 60∼75% VO_2peak_×60 min RE: the former 4 weeks: 60% *1RM; 8∼12 repetitions/set; 1∼2 sets the latter 8 weeks: 60% *1RM; 8–12 repetitions/set; 2 sets until fatigue	AE: 60 min/session; 3 sessions/week RE: 60 min/session; 3 sessions/week	3 months	AE: treadmills, ellipticals, or stationary bikes RE: 10 whole-body exercises using stack weight equipment (leg press, leg extension, leg flexion, chest press, latissimus pull down, seated row, biceps curl, and triceps extension) + push-ups and sit-ups CON: no exercise	AE and RE and CON: all groups were asked to follow a weight maintenance diet (55–60% carbohydrate, 15–20% protein, and 25–30% fat)	visceral fat (kg)
5	Staiano et al. (2017)	America	adolescents (age:14–18 years; BMI ≥85th percentile; gender: female)	AE: 18 CON: 15	AE: self-select intensity level	AE: 60 min/session; 3 sessions/week	12 weeks	AE: dance exergaming CON: no exercise	none	VAT (kg)
6	Barbeau et al. (2007)	America	children (age:8–12 years; BMI ≥85th percentile; gender: female)	AE: 118 CON: 83	AE: HR > 150 bpm	AE: 80 min/session (25 min skills instruction, 35 min aerobic PA, and 20 min toning/stretching); 5 sessions/week	10 months	AE: MVPA games (basketball, tag, softball, relay races) CON: no exercise	none	VAT (cm^3^)
7	Dias et al. (2018)	Australia	children and adolescents (age: 7–16 years; BMI ≥30 kg/m^2^; gender: male and female)	AE: 17 HIIT: 15 CON: 14	AE: 60∼70% HR_max_ HIIT: high-intensity: 85∼95% HR_max_×4 min; interval: 50∼70% HR_max_×3 min	AE: 44 min/session; 3 sessions/week HIIT: 40 min/session; 3 sessions/week	12 weeks	AE and HIIT: walking or running on a treadmill or cycling on a stationary bike CON: no exercise	AE and HIIT and CON: all groups received nutrition advice	VAT volume (cm^3^)
8	Zhang et al. (2021)	China	young adults (age:18–23 years; BFP ≥30%; gender: female)	AE: 11 HIIT: 12 SIT_all-out_: 11 SIT _120_: 12 CON: 13	AE: 60% VO_2peak_ HIIT: high-intensity: 90% VO_2peak_ ×4 min; interval: 3 min SIT _all-out_: high-intensity: all-out sprint×6 s; interval: 9 s SIT _120_: high-intensity: 120% VO_2peak_×1 min; interval: 1.5 min	AE and HIIT and SIT _all-out_ and SIT _120_: the former 4 weeks: 1 session/day; 3 days/week the latter 8 weeks: 1 session/day; 4 days/week	12 weeks	AE and HIIT and SIT _120_: electronically braked cycle ergometer SIT _all-out_: Monark Wingate cycle ergometer CON: no exercise	AE and HIIT and SIT _all-out_ and SIT _120_ and CON: all participants recorded their daily food intake. Dietary advice was provided whenever violation of maintenance of daily caloric intake was detected	visceral fat area (cm^2^)
9	Mitchell et al. (2002)	America	adolescents (age:13∼16 years; TSF ≥85th percentile; gender: male and female)	AE: 20 CON: 15	MIAE: 55–60% VO_2peak_ HIAE: 75–80% VO_2peak_	MIAE: 43 min/session; 5 sessions/week HIAE: 29 min/session; 5 sessions/week	8 months	MI and HIAE: treadmills, bicycles, rowers, and stair-steppers, aerobics, basketball, badminton, kickball, and aerobic slide CON: no exercise	AE and CON: all participants received the information about nutrition	VAT (cm^3^)
10	Davis et al. (2011)	America	adolescents (age:14–18 years; BMI ≥85th percentile; gender: female)	CE: 14 CON: 12	CE: 70∼85% HR_max_	CE: 60∼90 min/session (AE×30–45min + RE×30–45 min); 2 sessions/week	16 weeks	CE: treadmill, elliptical machines, and aerobic classes + strength training exercises using weight machines (upper and lower body exercises were paired together) CON: no exercise	CE and CON: all participants’ dietary intake was assessed by 3-day diet records (two weekdays, one weekend day)	VAT (L)
11	Saelens et al. (2011)	America	children (age: 7–11 year; BMI ≥85th percentile; gender: male and female)	AE: 14 CON: 15	AE: not mentioned	AE: 90 min/session; 6 sessions/week	14 weeks	AE: MVPA CON: no exercise	AE and CON: all participants reduced calories	visceral fat (cm^3^)
12	Davis et al. (2012)	America	children (age: 7–11 years; BMI ≥85th percentile; gender: male and female)	low-does AE: 71 high-does AE: 73 CON: 78	low&high-dose AE: average HR > 150 beats/min	low-does AE: 20 min/session; 5 sessions/week high-does AE: 40 min/session; 5 sessions/week	13 weeks	low and high-dose AE: running games, jump rope, and modified basketball and soccer CON: no exercise	low and high-dose AE and CON: all families enrolled were offered monthly lifestyle education classes that addressed topics such as healthy diet	visceral fat (cm^3^)
13	Zhang et al. (2017)	China	young adults (age:18–22 years; BMI ≥ 25 kg/m^2^; gender: female)	AE: 15 HIIT: 15 CON: 13	AE: 60% VO_2peak_ HIIT: high-intensity: 90% VO_2peak_×4 min; interval: 3 min	AE and HIIT: the former 4 weeks: one session/day; 3 days/week the latter 8 weeks: one session/day; 4 days/week	12 weeks	AE: cycle ergometer HIIT: cycling exercise bouts CON: no exercise	AE and HIIT and CON: all participants recorded their daily food intake. Dietary advice was provided to the participants by the dietician if the maintenance of the weekly caloric intake was violated	visceral fat area (cm^2^)
14	Tong et al. (2018)	China	young adults (age:18–23 years; BFP ≥30%; gender: female)	HIIT: 16 SIT: 16 CON: 14	HIIT: high-intensity: 90% VO_2peak_×4 min; interval: 3 min SIT: high-intensity: all out sprint×6 s; interval: 9 s	HIIT and SIT: the former 4 weeks: one session/day; 3 days/week the latter 8 weeks: one session/day; 4 days/week	12 weeks	HIIT and SIT: cycle ergometer CON: no exercise	HIIT and SIT and CON: all participants recorded their daily food intake. Dietary advice was provided to the participants by the dietician if the maintenance of the weekly caloric intake was violated	visceral fat area (cm^2^)
15	Cao et al. (2022)	China	adolescents (age:11–13 years; BMI ≥95th percentile; gender: male)	AE: 11 HIIT: 12 CON: 13	AE: 60∼70% MAS HIIT: high-intensity: 90–100% MAS×15 s; interval: 50% MAS×15 s	AE: 30∼40 min/session; 3 sessions/week HIIT: 30–40 min/session; 3 sessions/week	12 weeks	AE and HIIT: running on an outdoor track CON: no exercise	none	VAT (g)

Abbreviations: CON, control group; ML, maximum load; HRmax, maximum heart rate; MIAE, moderate-intensity AE; HIAE, high-intensity AE; MVPA, moderate to vigorous physical activity; BFP, body fat percentage; SIT, sprint interval training; SIT all-out all-out sprint interval training; SIT 120, supramaximal sprint interval training; MAS, maximal aerobic speed; TSF, triceps skinfold thickness.

Of these 15 articles, thirteen (87%) involved the isolated AE intervention, three (20%) involved the isolated RE intervention, three (20%) involved the CE intervention and five (33%) involved the HIIT intervention. For studies related to AE, nine (69%) studies designed running or walking as an AE intervention. Six (46%) studies designed cycling as an AE intervention. Only one (8%) study conducted dancing as an AE intervention and the other three (23%) studies designed several ball games like basketball, softball, badminton, and kickball as AE interventions. For studies related to RE, all studies (*n* = 3, 100%) performed the whole-body muscle strength training (e.g., leg press, leg extension, leg flexion, chest press, latissimus pulldown, seated row, bicep curl, and triceps extension) with weight machines as RE interventions. For the studies related to CE, all of them (*n* = 3, 100%) combined the AE and RE modalities mentioned above. Of the five studies involving HIIT, three conducted the cycling exercise as an intervention. And one study designed walking or running on a treadmill as well as cycling on a stationary bike as interventions. One study designed running on an outdoor track as an intervention.

**FIGURE 2:**
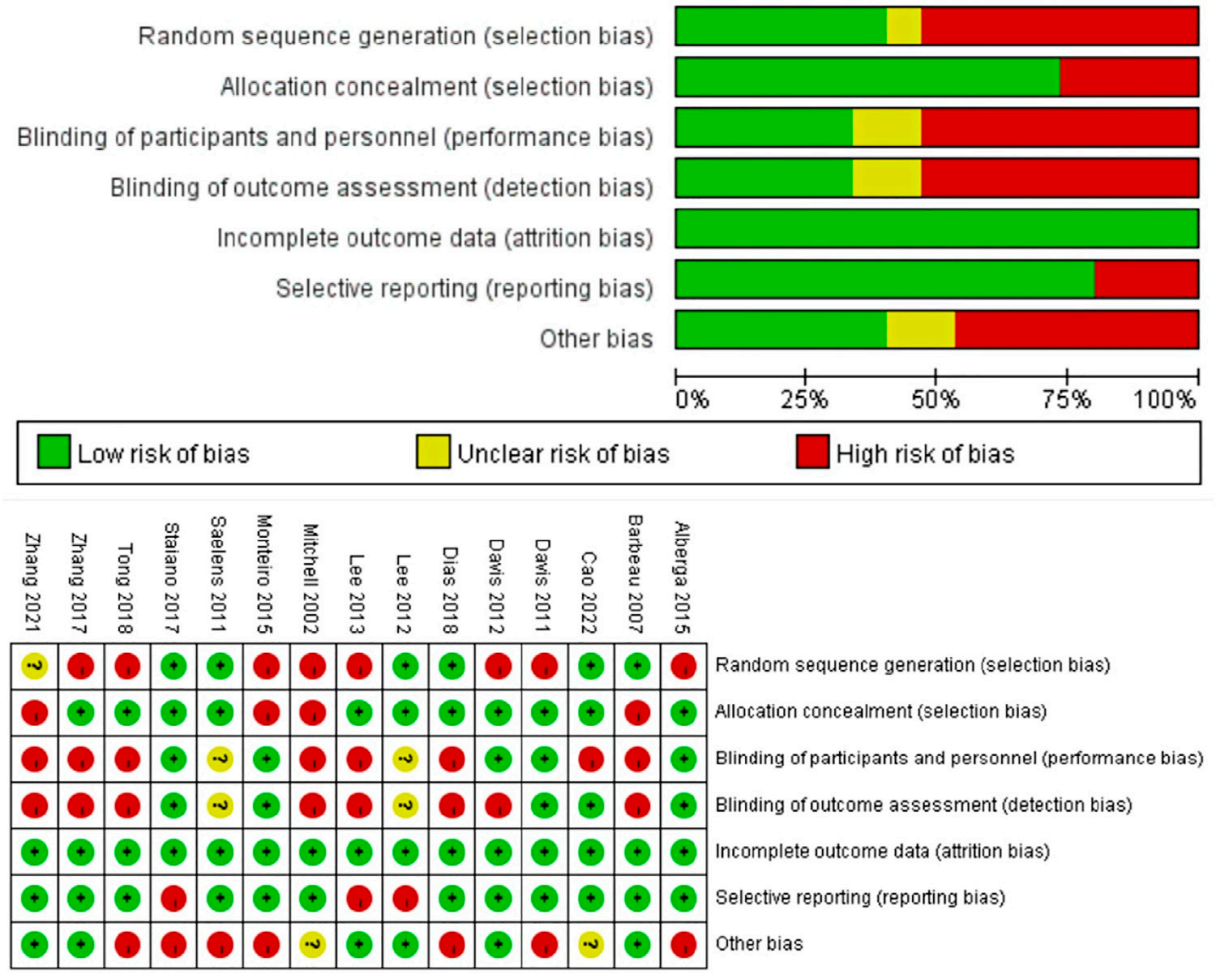
The bias of the included studies.

According to the instruction developed by Higgins (Higgins et al., 2011), we evaluated the quality of included articles. The bias of included studies mainly came from the random sequence generation (selection bias), the blinding of participants and personnel (performance bias), and the blinding of outcome assessment (detection bias) (shown in Figure 2).

### Effect of exercise interventions on visceral fat

**FIGURE 3:**
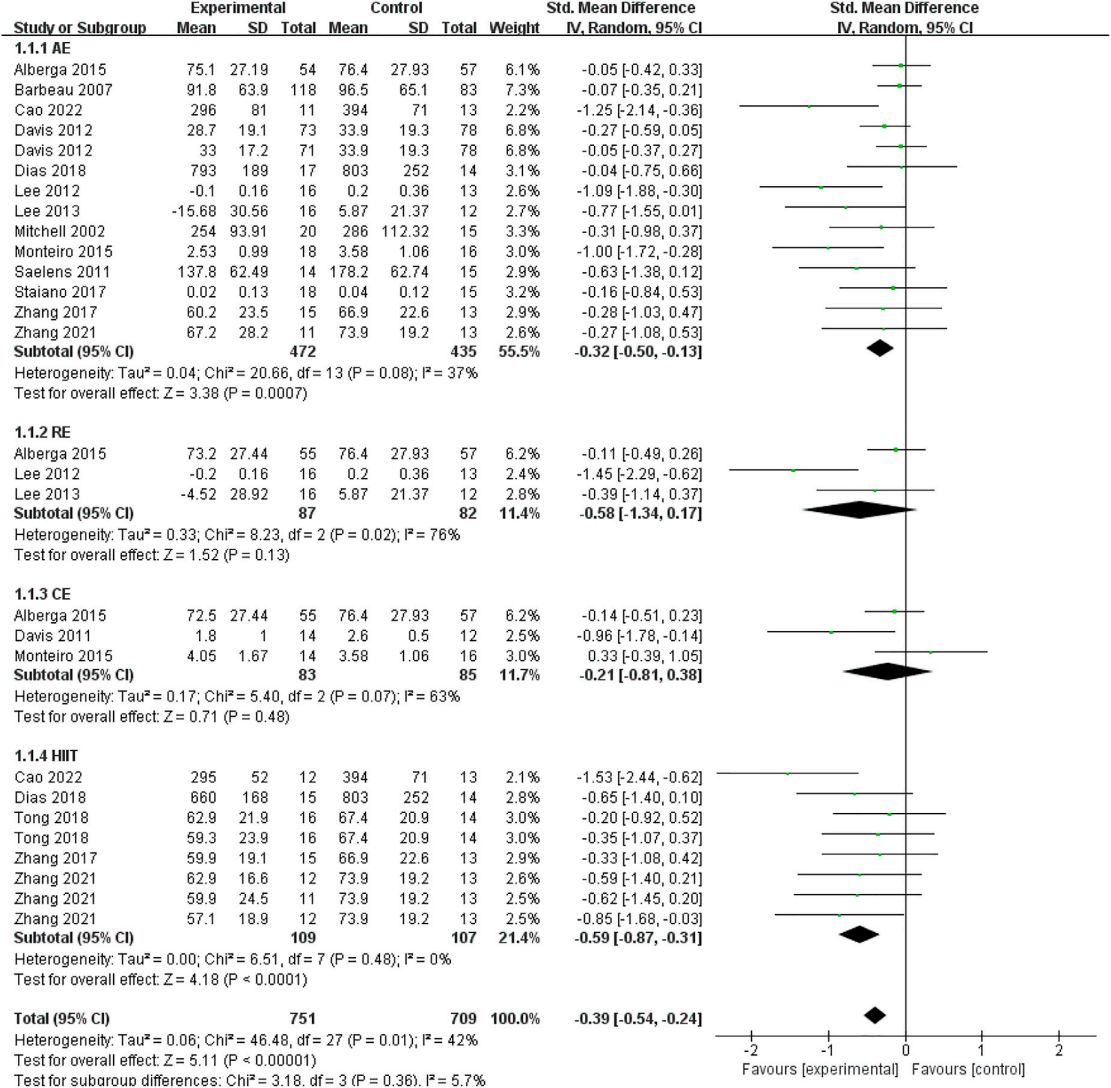
The effect of different exercise interventions on visceral fat.

A total of 28 data points from fifteen studies presented the influence of exercise interventions on visceral fat in children, adolescents, and young adults with overweight or obesity. As shown in Figure 3, there was a significant difference between the exercise intervention and control groups based on a random-effect model (SMD = −0.39; 95% CI = −0.54 to −0.24; *p* < 0.00001; I^2^ = 42%), which indicated that exercise interventions had a significant effect on reducing visceral fat in obese or overweight young individuals. To compare the effectiveness of different exercise interventions on the decline of visceral fat, we made a subgroup analysis based on the exercise types (i.e., AE, RE, CE, HIIT). Fourteen data points from thirteen studies demonstrated the effectiveness of AE on visceral fat in young individuals. As shown in Figure 3, there was a significant difference between the AE and control groups (SMD = −0.32; 95% CI = −0.50 to −0.13; *p* = 0.0007; I^2^ = 37%). It indicated that AE was an effective intervention for young individuals to decrease visceral fat. For RE, three data points from three studies were involved in this meta-analysis. No significant difference was found between the RE and control groups (SMD = −0.58; 95% CI = −1.34 to 0.17; *p* = 0.13; I^2^ = 76%). It implied that RE had a non-significant effect on the decline of the visceral fat in obese or overweight young people. For CE, three data points from three studies reported the effectiveness of the combination of AE and RE interventions on obese or overweight subjects’ visceral fat. As presented in Figure 3, no significant difference was found between the CE and control groups (SMD = −0.21; 95% CI = −0.81 to 0.38; *p* = 0.48; I^2^ = 63%). It suggested that CE couldn’t help young individuals decrease visceral fat. For HIIT, eight data points from five studies were used to report the effectiveness of HIIT on the visceral fat of young individuals with obesity. As shown in Figure 3, there was a significant difference between the HIIT and control groups (SMD = −0.59; 95% CI = −0.87 to −0.31; *p* < 0.0001; I^2^ = 0%), which implied that HIIT was significantly effective for obese or overweight young individuals to reduce visceral fat. Based on the effect size in AE (SMD = −0.32) and HIIT (SMD = −0.59), the effect of HIIT on reducing the visceral fat appeared to be more obvious than that of AE.

### Effect of exercise interventions on visceral fat in overweight/obese young individuals with different ages

**FIGURE 4:**
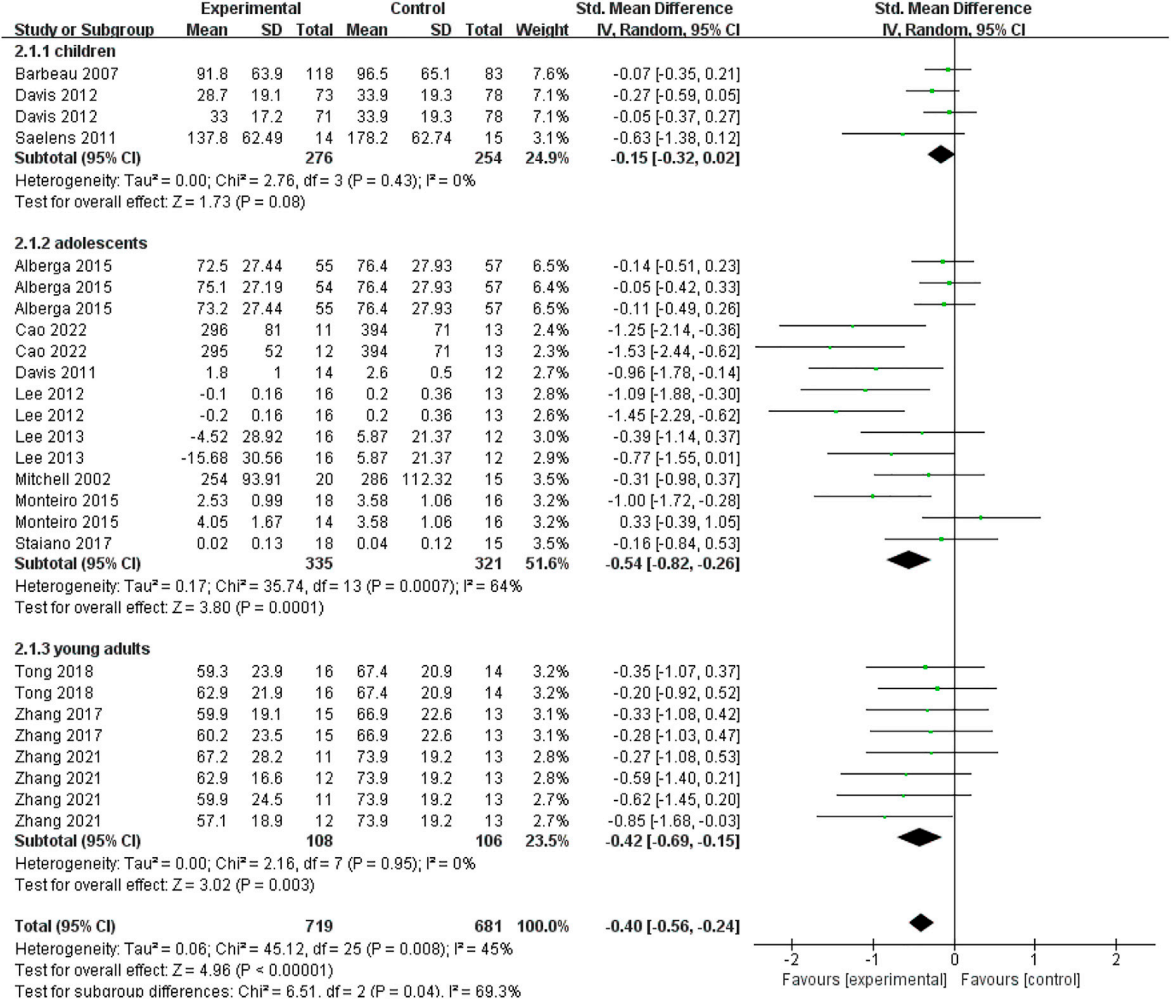
Subgroup analysis of age related to the effect of exercise interventions on visceral fat in young individuals with obesity or overweight.

As shown in Figure 4, to compare the effects of exercise interventions on visceral fat consumption in different age groups, we made a subgroup analysis based on the subjects’ age (i.e., children aged 6–12 years old, adolescents aged 12–18 years old, young adults aged 18–24 years old). Four data points from three studies evaluated the effectiveness of exercise interventions on visceral fat in overweight or obese children aged 6–12 years old. For children (6–12 years old) group, no significant difference between the exercise and control groups (SMD = −0.15; 95% CI = −0.32 to 0.02; *p* = 0.08; I^2^ = 0%) was found, which proposed that exercise interventions couldn’t help obese children burn the visceral fat. For adolescents aged 12–18 years old, fourteen data points from eight studies were pooled in our meta-analysis. As shown in Figure 4, for adolescents (12–18 years old) group, there was a significant difference between experimental and control groups based on a random-effect model (SMD = −0.54; 95% CI = −0.82 to −0.26; *p* = 0.0001; I^2^ = 64%). It indicated that exercise interventions were conducive to decreasing visceral fat in adolescents with obesity or overweight. For young adults aged 18–24 years old, eight data points from three studies reported the effectiveness of exercise interventions on visceral fat consumption. Figure 4 showed that there was a significant difference between experimental and control groups (SMD = −0.42; 95% CI = −0.69 to −0.15; *p* = 0.003; I^2^ = 0%), which implied that the exercise could significantly decrease young adults’ visceral fat.

### Effect of exercise interventions on visceral fat in overweight/obese young individuals with different genders

**FIGURE 5:**
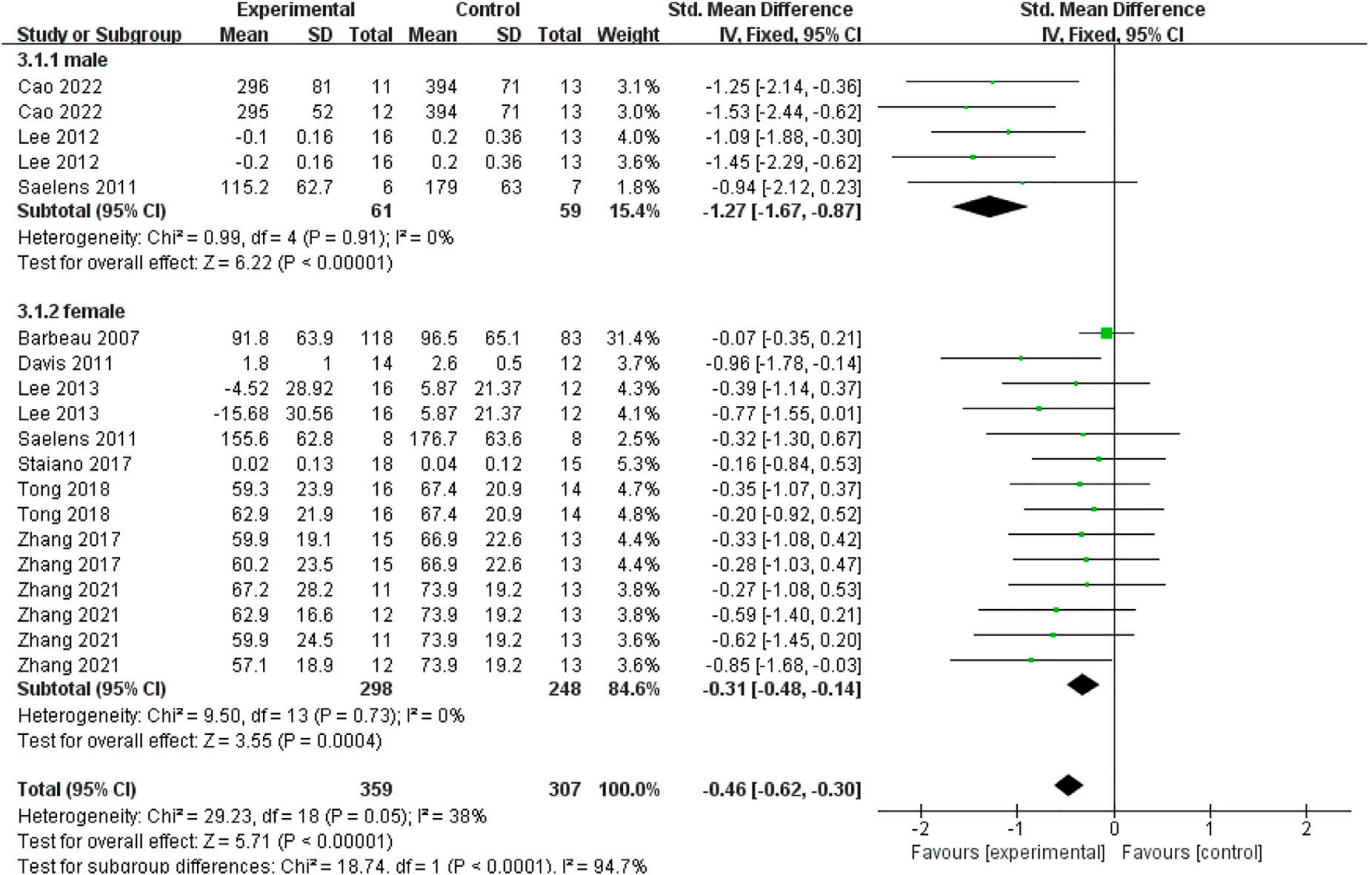
Subgroup analysis of gender related to the effect of exercise interventions on visceral fat in young individuals with obesity or overweight.

A total of nineteen data points from ten studies reported the specific genders of subjects. To distinguish the gender difference in the effect of exercise interventions on visceral fat in young individuals, we conducted a subgroup analysis based on the genders of young participants. As shown in Figure 5, for males, there was a significant difference between experimental and control groups based on a fixed-effect model (SMD = −1.27; 95% CI = −1.67 to −0.87; *p* < 0.00001; I^2^ = 0%). For females, there was also a significant difference between experimental and control groups based on a fixed-effect model (SMD = −0.31; 95% CI = −0.48 to −0.14; *p* = 0.0004; I^2^ = 0%). It indicated that exercise interventions could significantly decrease the visceral fat in both young males and females with overweight or obesity. Based on the effect size in different genders, the decreased effect of exercise interventions on visceral fat was more obvious in male subjects (SMD = −1.27) than that in female subjects (SMD = −0.31).

## Discussion

In this meta-analysis, the evidence from 15 studies regarding the effects of AE, RE, CE, and HIIT was summarized. This synthesis has suggested that the exercise is highly associated with a reduction in visceral fat of obese or overweight young individuals aged 6–24 years old. Among the exercise interventions included in our study, the effectiveness of AE and HIIT is significantly positive for reducing the visceral fat in young people with obesity. And the effect of HIIT seems better than AE. However, the effect of RE and CE on visceral fat was not statistically significant. For different age groups, the effect of the exercise on visceral fat consumption was significant in adolescents (12–18 years old) and young adults (18–24 years old) with overweight or obesity, but not in children (6–12 years old). In addition, the results derived from the gender-based subgroup analysis illustrated that exercise interventions could decrease visceral fat in both males and females. And the effect of the exercise was more significant in males than that in females.

The results demonstrated that AE and HIIT had a significant effect on the reduction of visceral fat in young individuals with obesity. For AE, it has been suggested that AE can increase the secretion of catecholamines, which are regarded as the lipolytic hormone, by inducing sympathetic nervousness tension (Arner, 1999; Monda et al., 2019). Some studies found that the lipolytic effect of catecholamines on VAT was even more pronounced than the abdominal subcutaneous adipose (Ostman et al., 1979; Marin et al., 1992). This may partly explain that, in our meta-analysis, AE is helpful for young individuals to lose visceral fat. Another effective exercise intervention identified in this meta-analysis was HIIT. On the one hand, it has been shown that HIIT also can stimulate visceral adipose lipolysis by facilitating catecholamine secretion (Lonnqvist et al., 1995; Trapp et al., 2007). On the other hand, HIIT can suppress appetite, which may decrease the energy intake and reduce visceral fat accumulation (Boutcher, 2011; Panissa et al., 2019). Moreover, we found that the effect of HIIT seemed even better than that of AE. This may be partly explained by that the higher intensity of HIIT rather than the moderate intensity of AE has been illustrated to be related to the release of more hormones (e.g., catecholamine, growth hormone) (Pritzlaff et al., 2000; Trapp et al., 2007), which can accelerate adiposity oxidation and decrease visceral fat (Stanley and Grinspoon, 2015). However, an original study found a similar effect of 6-week moderate-intensity continuous training and HIIT on visceral fat size when the energy expenditure of exercise bout was equal (Gerosa-Neto et al., 2019). The same result wasn’t obtained in this review, because our meta-analysis could not uniformly control the energy of all the included studies.

This meta-analysis indicated that RE and CE had non-effective influence on decreasing visceral fat for young individuals with obesity. For RE, the non-significant effect of the exercise may be due to the different proportions of the energy supply system and different energy substrates (Moghetti et al., 2016). It is known that the energy of the RE is mainly supplied by the anaerobic energy system. Consequently, the adenosine triphosphate (ATP), creatine phosphate (CP), and muscle glycogen rather than the fat are mainly consumed to provide the energy during the resistance training. For CE, it is widely documented that the long-term and low-intensity AE is easier to mobilize fat for the energy supply (Turcotte et al., 1992; Kiens et al., 1993; Ranallo and Rhodes, 1998). However, when the AE is combined with the RE, the decrease in the proportion of AE during the exercise period may illustrate the reason why CE failed to burn visceral fat in young individuals with overweight or obesity. Additionally, the small sample size (*n* = 83, 7%) might cause the bias.

Our meta-analysis revealed that exercise interventions could effectively decrease the visceral fat in adolescents aged 12–18 years old and young adults aged 18–24 years old, but not in 6–12-year-old children. For adolescents and young adults, the significant effect of the visceral fat decline could be interpreted as that they may have a better understanding of the technical movements during the exercise and the level of the exercise completion is higher. For children, a study suggested that children including obese or overweight children had less visceral fat in the abdomen area (Staiano et al., 2013). Hence, the exercise may have a more obvious effect on decreasing subcutaneous fat rather than visceral fat in low-aged obese children.

For subjects in different gender groups, we found that exercise interventions were more helpful to reduce visceral fat in young males than that in young females with obesity or overweight. This could be explained by the gender differences in hormone levels. Various studies have indicated that the estrogen can promote the proliferation of adipocyte precursors to produce new mature fat cells in the visceral region (Tchoukalova et al., 2010; Gavin and Bessesen, 2020). Conversely, during puberty, boys can secrete more androgens that stimulate lipolysis than girls (Blouin et al., 2008). Therefore, the dominance of androgens in boys has more tendency for the visceral fat removal. Additionally, we speculated that males were more compliant and interested in the exercise than females. So, females might not well finish the exercise in experiments due to a lack of interest in the exercise. On the other hand, males might spend more time exercising in their daily lives out of experiments. In contrast, females often conducted sedentary behavior. Win et al. have also suggested that females exercised less regularly than males (prevalence rate = 0.63, 95% CI = 0.51–0.76) (Win et al., 2015).

Although this study demonstrated the outstanding effects of AE and HIIT on visceral fat in young individuals with obesity, there are some limitations. First, it is worth mentioning that the outcomes of this meta-analysis are restricted to the effects of various exercise types on the visceral fat of young people with obesity. Hence, the findings of our study do not apply to other benefits of exercise interventions for obese or overweight young individuals, such as the improved cardiorespiratory fitness, the increased muscle mass. The exercise interventions that have no apparent effects on reducing visceral fat in young individuals with obesity may have other health benefits. Second, except for age and gender, the effect of exercise interventions on visceral fat may vary from the degree of overweight (i.e., overweight or obesity) of young individuals. But several covered studies in our meta-analysis provided a mixed sample including overweight and obese individuals. This prevented us from extracting the independent data points from the mixed BMI or percentage of body fat group. Third, our study focused on comparing the experimental group (exercise) with the control group (no exercise). Thus, this meta-analysis did not consider the effect of diet on visceral fat. Future meta-analyses can investigate the interaction effect of the diet and the exercise on visceral fat. Last, the limited number of included articles (*n* = 15) may result in a certain degree of influence on the effect. Although this review and meta-analysis is systematic and rigorous, we did not include unpublished articles. This may affect the comprehensiveness of this study.

## Conclusion

This review and meta-analysis indicates that exercise interventions are effective in reducing visceral fat in young people with overweight and obesity, especially in adolescents (12–18 years old) and young adults (18–24 years old). Among different exercise types, AE and HIIT have a significant effect on decreasing visceral fat and HIIT appears to be the most effective exercise type. The effectiveness of exercise interventions on the decline of visceral fat is more significant in boys than that in girls.

## Data Availability

The original contributions presented in the study are included in the article/Supplementary Material, further inquiries can be directed to the corresponding author.
