# Erratum: Effects of different exercise types on visceral fat in young individuals with obesity aged 6–24 years old: A systematic review and meta-analysis

**DOI:** 10.3389/fphys.2022.1092794

**Published:** 2022-11-25

**Authors:** Frontiers Production Office

**Affiliations:** Frontiers Media SA, Lausanne, Switzerland

**Keywords:** exercise, visceral fat (VFA), young individuals, adolescents, obesity, overweight, meta-analysis

Due to a production error, two headings were removed in the Results section. The heading ‘Effect of exercise interventions on visceral fat in overweight/obese young individuals with different ages’ should be between line 894 and line 895 before the paragraph starting “As shown in Figure 4…”. The heading ‘Effect of exercise interventions on visceral fat in overweight/obese young individuals with different genders’ should be between line 951 and 952 before the paragraph starting “A total of nineteen data points…”

The publisher apologizes for the mistake. The original article has been updated.

